# Meta-analysis based on weighted ordered P-values for genomic data with heterogeneity

**DOI:** 10.1186/1471-2105-15-226

**Published:** 2014-06-28

**Authors:** Yihan Li, Debashis Ghosh

**Affiliations:** 1 Department of Statistics, Pennsylvania State University, University Park, Pennsylvania 16802, USA

**Keywords:** Fisher’s combined probability test, Meta-analysis, Ordered p-values, Weighted order statistic

## Abstract

**Background:**

Meta-analysis has become increasingly popular in recent years, especially in genomic data analysis, due to the fast growth of available data and studies that target the same questions. Many methods have been developed, including classical ones such as Fisher’s combined probability test and Stouffer’s Z-test. However, not all meta-analyses have the same goal in mind. Some aim at combining information to find signals in at least one of the studies, while others hope to find more consistent signals across the studies. While many classical meta-analysis methods are developed with the former goal in mind, the latter goal has much more practicality for genomic data analysis.

**Results:**

In this paper, we propose a class of meta-analysis methods based on summaries of weighted ordered p-values (WOP) that aim at detecting significance in a majority of studies. We consider weighted versions of classical procedures such as Fisher’s method and Stouffer’s method where the weight for each p-value is based on its order among the studies. In particular, we consider weights based on the binomial distribution, where the median of the p-values are weighted highest and the outlying p-values are down-weighted. We investigate the properties of our methods and demonstrate their strengths through simulations studies, comparing to existing procedures. In addition, we illustrate application of the proposed methodology by several meta-analysis of gene expression data.

**Conclusions:**

Our proposed weighted ordered p-value (WOP) methods displayed better performance compared to existing methods for testing the hypothesis that there is signal in the majority of studies. They also appeared to be much more robust in applications compared to the *r*th ordered p-value (rOP) method (Song and Tseng, Ann. Appl. Stat. 2014, 8(2):777–800). With the flexibility of incorporating different p-value combination methods and different weighting schemes, the weighted ordered p-values (WOP) methods have great potential in detecting consistent signal in meta-analysis with heterogeneity.

## Background

Meta-analysis has long been used to integrate data and/or results from multiple studies targeting the same questions. It is commonly used in many areas of statistical applications such as clinical studies and psychology experiments. In recent years, meta-analysis has been frequently adopted in genomic data analysis, due to the fast development of high-throughput technology and the vast amounts of data available in public databases.

Many meta-analysis methods have been developed throughout the years. Roughly speaking, there are two main approaches to meta-analysis methods Song and Tseng [1]. The first directly combines the p-values from the studies, while the second attempts to model the data or the effect sizes from the combined studies. The former includes methods such as the Fisher’s combined probability test [2] and the Stouffer’s Z-test [3], as well as weighted variations of these classical tests. The latter includes a variety of fixed effects and random effects models, such as GeneMeta [4]. Each approach has its advantages and disadvantages. The p-value combining methods are relatively flexible in that they require minimal information and assumptions from the studies. In this paper we will mainly focus on methods that directly combine the p-values.

Most of the traditional meta-analysis methods (e.g. Fisher’s and Stouffer’s methods) aim at testing the alternative hypothesis that at least one of the studies is non-null. While this aligns with earlier goals of meta-analyses to gain power in detecting signals by combining multiple studies, it is frequently not the case with genomic data. In meta-analysis of genomic studies for example, the goal is often to identify genes that are differentially expressed in a consistent pattern across multiple studies. The extreme of this would be to test for the alternative that the null can be rejected for all the studies. A solution to this extreme alternative dates back to the maxP method by Wilkinson [5]. However, the maxP method is often considered too conservative. A recent approach by Phillips and Ghosh [6] improves the power of testing for this disjunction of nulls when the rejection of all p-values associated with a gene is required. In practical meta-analyses, the goal of rejecting all studies may be considered too extreme. Ideally, we would want to target at detecting consistent signals across studies while avoiding being overly exclusive. This issue has gained attention in recent years, and a number of authors have tried to address this problem. Benjamini and Heller [7] discussed a framework for testing partial conjunction hypotheses, where they test for the alternative that at least *u* out of *n* null hypotheses are false against the partial conjunction null that no more than *n*−*u*+1 of the null hypotheses are true. Song and Tseng [1] proposed the *r*th ordered p-value (rOP) method that aims at testing the alternative hypothesis that there is signal in at least a given percentage of studies. Other methods exist that address this problem from different approaches, such as RankProd by Hong et al. [8] that looks for consistently highly ranked genes, and a weighted approach by Li and Ghosh [9] that weights genes by its expression consistency across studies.

We consider the problem of detecting signals in the majority of studies. Our approach adopts one aspect of the rOP method Song and Tseng [1] in that we also consider ordered p-values. But instead of using a single *r*th ordered p-value as the statistic (as rOP does), we combine all or a subset of the ordered p-values while weighting them based on their order (weighted ordered p-values, WOP). P-values closer to the median are highly weighted and the smallest/largest p-values are down-weighted. The idea is that among the collection of p-values, the median p-values are likely to be a better reflection of the behavior of the majority of studies than the smallest or largest p-values. Olkin and Saner [10] discussed a trimmed Fisher’s procedure that leaves out a number of the smallest and/or largest p-values from the calculation of Fisher’s statistic to remove the effect of possible aberrant extremes. In our consideration, we still keep the smallest/largest p-values because they do carry certain information, but we down-weight them because they may be relatively less relevant when considering the “majority” of studies. To reflect the up-weighting of the medians and down-weighting of the extremes, we calculated our weights based on the binomial distribution. To summarize the weighted ordered p-values, we mainly considered Fisher’s statistic and Stouffer’s Z-test. We also explored some other statistics, such as generalized Fisher’s test by Lancaster [11]. In general, other summary statistics can be used under this framework as well.

While many weighted variations of Fisher’s statistic and Stouffer’s statistic have been developed throughout the years, most of them distribute weights according to the sample sizes and/or effect sizes of the studies, or other similar considerations (e.g. Mosteller and Bush [12], Won et al. [13], Makambi [14]). Li and Tseng [15] proposed an interesting adaptively weighted statistic, where the weights are used to maximize the significance of the summary statistic. However, to the best of our knowledge, none of the weighting schemes are based on the ordered p-values, which is what makes our method unique. Xie et al. [16] discussed a meta-analysis approach using confidence distributions, where they incorporated the use of medians and kernel functions, but their approach is under a completely different framework from ours.

By incorporating more than one order statistic into our summary statistic, our method can be considered an expansion of the *r*th ordered p-value (rOP) method Song and Tseng [1]. In general, both the rOP method and the original summary statistic we use (e.g. Fisher’s or Stouffer’s method) are special cases under the WOP framework - one having all the weight on a single ordered p-value and the other having evenly distributed weights. However, our WOP methods have respective advantages over both the traditional summary statistics and the rOP method. Compared to the traditional summary statistics, the WOP methods better focus on detecting signal in a majority of studies. On the other hand, the WOP methods appear to be more robust compared to the rOP method. These observations are based upon results from simulation studies as well as data applications.

## Methods

### Hypothesis settings for meta-analysis

Before performing any meta-analysis, it is always important to figure out the goal of combining multiple studies. When a single hypothesis test is conducted, it is clear what the null and alternative hypotheses are. In meta-analysis we usually combine studies designed to test the same set of null and alternative hypotheses. However, the null and alternative hypotheses of the meta-analysis test are not always obvious and largely depends on the researcher’s goals. Here we consider the example of meta-analysis of differential expression studies of genomic data. Sometimes a gene is of interest as long as it is differentially expressed in at least one study, while other times we hope to target genes that are differentially expressed in all studies. Li and Tseng [15] and Song and Tseng [1] both provided extensive discussions on the different scenarios that lead to different hypothesis settings. Following their notation, let *θ*_*g* *k*_ be the true effect size for gene *g* (1≤*g*≤*G*) in study *k* (1≤*k*≤*K*). As in Song and Tseng [1], for a given 1≤*r*≤*K*, a general hypothesis setting for the meta-analysis of the *K* studies for a given gene *g* can be formulated as:

$$\;{\text{HS}}_{r}\;:\;\;\left\{\;H_{0}\;:\;\overset{K}{\underset{k=1}{\sum }}I(\theta_{\text{gk}}\;\neq \;0)\;=\;0\;\text{versus}\;H_{a}^{\left(r\right)}\;:\;\overset{K}{\underset{k=1}{\sum }}I(\theta_{\text{gk}}\;\neq \;0)\geq r\right\}\;\;.$$

When *r*=1, *HS*_1_ is the classical setting of testing for non-zero effect size in at least one study against the conjunction of nulls. *HS*_1_ is the hypothesis setting that Fisher’s method, Stouffer’s Z-test and many other traditional methods test for. When *r*=*K*, *HS*_*K*_ tests for the alternative that all the studies have non-zero effect size. For instance, the maxP method [5] tests for *HS*_*K*_. When 1<*r*<*K*,*HS*_*r*_ provides a compromise between the two aforementioned hypothesis settings, and tests for at least a pre-specified number of non-zero effects. For a given *r*, the rOP (*r*th ordered p-value) method Song and Tseng [1] is used to test for *HS*_*r*_.

In this paper, we test for non-zero effect sizes in a majority of studies against the null that the effects sizes are zero in all studies. Thus we are testing against the conjunction of nulls while trying to focus on a certain subset of the non-null space. In general, the hypothesis for our meta-analysis approach can be considered to fall under the *HS*_*r*_ setting. Song and Tseng [1] suggested a few data-driven methods for selecting *r*. To prevent any potential issues of post hoc choices of *r*, we choose to fix *r* before any analysis is conducted. While it is hard to specify what the term “majority” exactly means, as a general rule, we choose to test the hypothesis setting *HS*_*m*_, where *m*=⌈*K*/2⌉, ⌈*x*⌉ being the smallest integer no less than *x*. Essentially we are targeting the alternative that at least half of the studies have non-zero effect sizes. Under our weighted ordered p-values (WOP) framework, we are able to develop methods for other hypothesis settings with *r* ranging from *m*+1 to *K*. But in general we hope to provide a simple to use method without having to put too much effort in selecting a particular *r*, and therefore we will focus mostly on testing *HS*_*m*_. We will later show through data applications that using our WOP methods for testing *HS*_*m*_ provides more robust results compared to using the rOP method with different choices of *r*.

### A framework for weighted ordered P-values (WOP) methods

We first describe the general framework for the weighted ordered p-values (WOP) methods. Suppose we have p-values *p*_1_,*p*_2_,⋯,*p*_*K*_ for testing the hypothesis of interest for each of the *K* studies. Let *p*_(1)_,*p*_(2)_,⋯,*p*_(*K*)_ be the ordered p-values. Now consider a set of weights *w*_1_,*w*_2_,⋯,*w*_*K*_ associated with the corresponding ordered p-values. Summary statistics of weighted ordered p-values can be expressed in the following general form:

$$T=\overset{K}{\underset{i=1}{\sum }}w_{i}H(p_{\left(i\right)}).$$

As mentioned by previous authors, many traditional p-value combination methods can be expressed in the general form of $T^{\prime }=\sum_{i=1}^{K}w_{i}^{\prime }H(p_{i})$ (for example, see Zaykin [17]). For instance, Stouffer’s Z-test takes *H*(·) to be the inverse normal function, while Fisher’s method has *H*(*p*_*i*_)=−2 log(*p*_*i*_). The difference between *T*^′^ and *T*, albeit subtle in notation, is the essence of the WOP framework. In the WOP framework, the weight *w*_*i*_ is associated with the *i*th ordered p-value *p*_(*i*)_. In other words, the ranking of a p-value in relation to the p-values from the other studies determines its weighting. In traditional weighted p-value combining methods, the weight assigned to a p-value is associated with the characteristics of that particular study, be it the sample size, the effect size, or other features.

Allowing the weights to depend on the ordering of the p-values opens up a whole new area of considerations when combining multiple p-values. We can consider giving more weights to the p-values that are closer to the center of the distribution of the p-values since they might hold more credibility, and at the same time down-weight the outlying p-values. For instance, if the entirety of weights is placed on the *r*th ordered p-value, the statistic reduces to the rOP method. However, using the WOP framework, if we highly weight the *r*th ordered p-value but still distribute some weight to the other p-values, the method can be viewed as a more robust version of the rOP method for testing *HS*_*r*_.

In this paper, we shall develop a few specific methods under the WOP framework. We consider weights based on the binomial distribution, which will be described in more detail in the next section. As for the p-value combining methods, we shall focus on Fisher’s method, with *H*_*F*_(*p*_(*i*)_)=−2 log(*p*_(*i*)_), and Stouffer’s method, with *H*_*Z*_(*p*_(*i*)_)=*ϕ*^−1^(1−*p*_(*i*)_), *ϕ*(·) being the standard normal distribution function.

### Binomial weights and half-binomial weights

In this section, we discuss two possible weighting schemes for the WOP framework. We mainly consider testing the alternative hypothesis that the effect sizes are non-zero in the majority of studies, which is the hypothesis setting *HS*_*m*_. We will also briefly discuss extending the weighting schemes to testing other hypothesis settings *HS*_*r*_, for *m*<*r*≤*K*.

Inspired by the rOP method, which uses the *r*th ordered p-value for testing the hypothesis setting *HS*_*r*_, we consider placing the highest weight on the median p-values for testing *HS*_*m*_. This makes intuitive sense, since if a consensus does exist among the studies, we have reason to believe that the behavior of the majority of studies should be best captured by the p-values that are closer to the center of the distribution. Since we do not insist on non-zero effect sizes for every single study, we consider down-weighting the largest p-values among the studies. On the other hand, p-value combining methods such as Fisher’s method are known to be very sensitive to single extremely small p-values, thus the smallest p-values should also be down-weighted, to avoid a small number of extremely small p-values biasing the results of the majority of studies. In summary, we would like our weighting scheme *w*_*i*_, as a function of *i*, to reflect a unimodal shape, with the highest weights being *w*_*m*_ (when *K* is odd) or *w*_*m*−1_ and *w*_*m*_ (when *K* is even), and such that *w*_*i*_ decreases as *i* goes to 1 or *K*.

To reflect the above properties of the weights, we constructed the weights based on the binomial distribution. Let *f*(*x*;*n*,*p*) be the probability mass function of the binomial distribution *B*(*n*,*p*), for *x*=0,1,⋯,*n*. We define the binomial weighting scheme such that

$$w_{i}^{b}=f(i-1;K-1,0.5),\;\;i=1,2,\cdots \;,\mathrm{K.}$$

In the binomial weighting scheme, all the weights are non-zero, thus every p-value contributes to the combined statistic. To further reduce the influence of the smallest p-values on the summary statistic, we may argue that only *p*_(*m*)_,*p*_(*m*+1)_,⋯,*p*_(*K*)_ matters in testing the alternative that at least *m* studies have non-zero effect size. With these considerations, we define what we call the half-binomial weighting scheme such that

$$w_{i}^{\text{hb}}=\left\{\begin{array}{cc} 0, & \;\;i=1,2,\cdots \;,m-1,\\ w_{i}^{b}, & \;\;i=m,m+1,\cdots \;,\mathrm{K.} \end{array}\right)$$

We will discuss more on the effects of these two different weighting schemes through simulation studies in later sections.

So far we have constructed the binomial weighting scheme and half-binomial weighting scheme for testing the hypothesis setting *HS*_*m*_. We can extend the ideas of these two weighting schemes to testing *HS*_*r*_, for *m*<*r*≤*K*. Instead of placing the highest weights on the medians, the highest weight can now be assigned to *p*_(*r*)_. When *r*≠*m*, we lose the natural symmetry of the weights. However, we can still base the weights on the binomial distribution. A few possible weighting schemes are defined below:

$$\;w_{i}^{b1}\;=\left\{\begin{array}{cc} 0, & \;\;i=1,\cdots \;,r-m\\ w_{i-(r-m)}^{b}, & \;\;i=r-m+1,\cdots \;,\mathrm{K.} \end{array}\right)$$

$$\;w_{i}^{b2}=f(i-1;K+2(r-m)-1,0.5),\;\;\;\;\;i=1,\cdots \;,\mathrm{K.}$$

$$\;w_{i}^{b3}\;=\;\;\left\{\;\begin{array}{cc} 0, & i=1,\cdots \;,2(r-m)\\ f(i-2(r-m)-1;K-2(r-m)\;-\;1,0.5), & i\;=\;2(r-m)+1,\cdots \;,\mathrm{K.} \end{array}\right)$$

**Figure 1:**
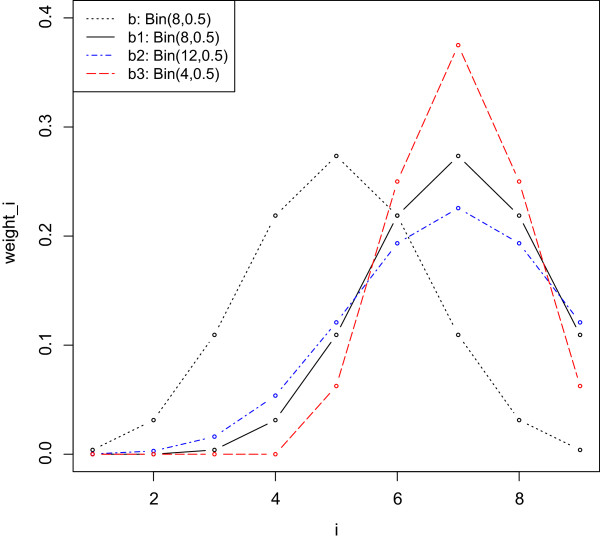
**Illustration of weighting schemes.** Intuition behind the three weighting schemes *w*^*b*1^, *w*^*b*2^ and *w*^*b*3^ for *m*<*r*≤*K*. The plot reflects an example of *K*=9. The weighting scheme *w*^*b*^ for testing *HS*_5_ is plotted as a reference. The other three weighting schemes are for testing *HS*_7_. From the plot we can easily see that *w*^*b*1^ is a direct shift of *w*^*b*^, which is based on the distribution of *B*(8,0.5). *w*^*b*2^ is based on the distribution of *B*(12,0.5), and *w*^*b*3^ is based on the distribution of *B*(4,0.5).

The weighting scheme *w*^*b*1^ is based on the same binomial distribution as *w*^*b*^, except that the values are shifted so that the center of the distribution falls on *r* instead of *m*. Because of the shift, the first few weights are set to be 0, while the last few values in the probability mass function of the binomial distribution are truncated. The weighting scheme *w*^*b*2^ increases the parameter *n* to *K*+2(*r*−*m*)−1 in the binomial distribution to ensure that the first few weights are non-zero. The weighting scheme *w*^*b*3^ decreases the parameter *n* to *K*−2(*r*−*m*)−1 in the binomial distribution so that the distribution is not truncated on the right. See Figure 1 for an illustration of the idea of these three weighting schemes. Corresponding half-binomial weights for these three binomial weighting schemes: *w*^*h* *b*1^, *w*^*h* *b*2^ and *w*^*h* *b*3^, can be easily constructed by setting the weights to be 0 for *i*=1,⋯,*r*−1.

### Implementation of the WOP methods

Under the null hypothesis, the *p*_*k*_’s are assumed to follow a uniform (0,1) distribution. For Stouffer’s Z-test, *H*_*Z*_(*p*_*i*_)=*ϕ*^−1^(1−*p*_*i*_) follows a standard normal distribution. Therefore the traditional weighted Z-test in the form of $\sum_{i=1}^{K}w_{i}^{\prime }H_{Z}(p_{i})$ still follows a normal distribution. For Fisher’s method, *H*_*F*_(*p*_*i*_)=−2 log(*p*_*i*_) has a chi-square distribution with two degrees of freedom. Therefore the distribution of the traditional weighted Fisher’s test $\sum_{i=1}^{K}w_{i}^{\prime }H_{F}(p_{i})$ is essentially weighted sums of exponential distributions. The distribution of weighted sums of exponential variables is not as straightforward, though many authors have researched on both the exact and approximations of this distribution, a summary of which can be found in Olkin and Saner [10].

When we consider weighted ordered p-values in the form of $\sum_{i=1}^{K}w_{i}H(p_{\left(i\right)})$, however, the problem becomes much more complicated. Even for Stouffer’s method, the distribution of the sum of weighted ordered normal variables is not readily available. As for Fisher’s method, Olkin and Saner [10] studied the distribution of the trimmed Fisher’s statistic, which is $\sum_{i=1}^{K}-2w_{i}\text{log}(p_{\left(i\right)})$ for the special case of *w*_*i*_=0 for *i*=1,⋯,*s*_1_ and *i*=*s*_2_,⋯,*K*. They transformed the distribution of the sum of ordered chi-squared variables back to a weighted sum of exponential variables without order. But as discussed in their paper, the exact distribution of weighted sums of exponential variables are generally impractical for practitioners, and we would therefore have to use approximation methods.

Considering the complexity of the exact distributions of weighted sums of ordered variables, as well as the fact that the uniformness of the original p-values is not always guaranteed in practice, we recommend two methods for obtaining the p-values for the WOP statistics: (1) permutation analysis, in the case that original data from all the studies are available; and (2) comparing to the numerical distribution, in the case that only the p-values for each gene and each study are known.

We first explain the steps of obtaining the WOP p-values through permutation analysis: 

•Let $T_{g}=\sum_{i=1}^{K}w_{i}H(p_{g(i)})$ denote the WOP statistic for gene *g*, where *p*_*g*(*i*)_ is the *i*th ordered p-value of *p*_*g*1_,⋯,*p*_*g* *K*_.

•Permute group labels in each study *B* times, and recalculate the p-values for the permuted data $p_{\text{gk}}^{\left(b\right)}$, for 1≤*k*≤*K*, 1≤*g*≤*G*, 1≤*b*≤*B*.

•Calculate the WOP statistics for the permuted p-values $T_{g}^{\left(b\right)}$ for 1≤*g*≤*G*, 1≤*b*≤*B*.

•The p-value for the WOP statistic *T*_*g*_ (1≤*g*≤*G*) is then computed as

$$p_{g}^{T}=\frac{\sum_{b=1}^{B}\sum_{g^{\prime }=1}^{G}I\{T_{g}\geq T_{g^{\prime }}^{\left(b\right)}\}}{B\cdot G}.$$

Once the p-values for the WOP statistics for each gene are obtained, we may apply the Benjamini-Hochberg (BH) method [18] on the $p_{g}^{T}$’s (1≤*g*≤*G*) to account for multiple testing across the genes and control the false discovery rate (FDR).

If the original data are not available, we can simulate the distribution of the WOP statistics numerically, by simulating *U*(0,1) random variables, the distribution of p-values under the null distribution. The WOP statistics calculated from the data can then be compared to the numerical distribution to obtain the WOP p-values. We simulated numerical distributions of the WOP statistics for testing *HS*_*m*_ based on the Fisher’s and Stouffer’s combination methods with binomial and half-binomial weighting schemes respectively, for study numbers ranging from 4 to 23. We conducted simulation studies to compare the WOP p-values obtained either though comparing with the numerical distribution or by performing permutation analysis. Results show that the two methods provide perfectly correlated p-values and that the number of rejections obtained from the two methods after applying the Benjamini-Hochberg adjustment are very similar (data not shown). Therefore both methods are reliable choices for obtaining the WOP p-values in practice. The numerical distribution provides an option for when the original data are not available and is also more time-efficient. The permutation analysis can be used if the uniformness of the original p-values are questionable but that the original data is available.

### Considerations of one- or two-sided tests

Previously we used two-sided alternatives as an example when setting up the hypothesis. The hypothesis setup *HS*_*r*_ can be similarly developed for one-sided alternatives. In fact, the interpretation of the meta-analysis results is easier for one-sided tests, since we do not need to worry about the concordance of the directions of the effect sizes as we do for two-sided tests. Since the WOP methods directly combine the p-values, the direction of the effect sizes are not taken into account for two-sided tests. Thus a significant result from the WOP meta-analysis of two-sided tests indicate that there are at least *r* studies with non-zero effect size, but without any implications about the concordance or discordance of the directions of the effects. This may not be an issue in the case that the direction of effect is not of great importance. However, in genomic data analysis, it is often desirable to distinguish between up- and down-regulated genes, and a result stating that the gene is differentially expressed across many studies but with possible opposite directions of expression change may be confusing. In these cases, it might be problematic to directly apply the WOP methods to the two-sided p-values. On the other hand, since both up- and down-regulated genes may be of interest at the same time, we cannot pre-specify one particular one-sided test for all genes. For such scenarios we recommend using the test of Pearson [19] in combination with the WOP methods. To do so, for each gene we need to conduct two WOP meta-analyses on one-sided p-values: one on the left-tailed p-values for all studies, and the other on the right-tailed p-values for all studies. Let $p_{\text{WOP}}^{L}$ and $p_{\text{WOP}}^{R}$ be the WOP meta-analysis p-values for the left-tailed and right-tailed tests respectively. We shall then adopt the idea of Pearson’s test and define $p_{\text{WOP}}^{C}=\min\{1,2\min(\overset{L}{\underset{\text{WOP}}{p}},\overset{R}{\underset{\text{WOP}}{p}})\}$, where the superscript “C” stands for “concordant”. As discussed in Owen [20], the equation for obtaining $p_{\text{WOP}}^{C}$ provides a conservative p-value for Pearson’s test. By adopting the Pearson’s test, the results are more interpretable. A significant result now indicates that the gene is consistently up- or down-regulated in at least *r* studies.

## Results and discussion

### A simulation study

We conducted a simulation study to compare the performances of the WOP methods with the original Fisher’s and Stouffer’s method, as well as with the rOP method Song and Tseng [1]. We shall also demonstrate the differences between the binomial and half-binomial weighting schemes through the simulation.

We simulate the setting of a meta-analysis of differential expression studies, with 2000 genes and 7 studies. Out of the 2000 genes, 1650 genes are assumed to be not differentially expressed in any study, while 50 genes are assumed to be differentially expressed in 1,2,⋯,7 studies respectively. The sample sizes for the treatment and control groups are randomly generated for each study, varying from 5 to 20. Gene expression values are randomly generated from normal distributions. Control samples are generated from a *N*(0,1) distribution, as well as treatment samples that are not differentially expressed. Treatment samples that are differentially expressed are generated from a *N*(1,1) distribution. Two-sample T-tests are used to obtain the p-values *p*_*g* *k*_ for each gene and each study. Our WOP methods aim at testing the hypothesis that the gene is differentially expressed in the majority of studies. In this case, *m*=4, corresponding to the hypothesis setting *HS*_4_. The rOP statistic Song and Tseng [1] for testing *HS*_4_ is the 4th ordered p-value. Note that the original Fisher’s and Stouffer’s method are supposed to test for *HS*_1_. We used permutation analysis to obtain the WOP p-values for binomial and half-binomial weighted Fisher’s and Stouffer’s statistic. P-values for the rOP method were also computed by permutation analysis as recommended in Song and Tseng [1]. P-values for the original Fisher’s and Stouffer’s method are computed directly via their respective distributions. To obtain a list of significant genes, the Benjamini-Hochberg procedure is applied to the p-values with the FDR controlled at the 0.05 level. Results are averaged over 100 replications.

**Figure 2:**
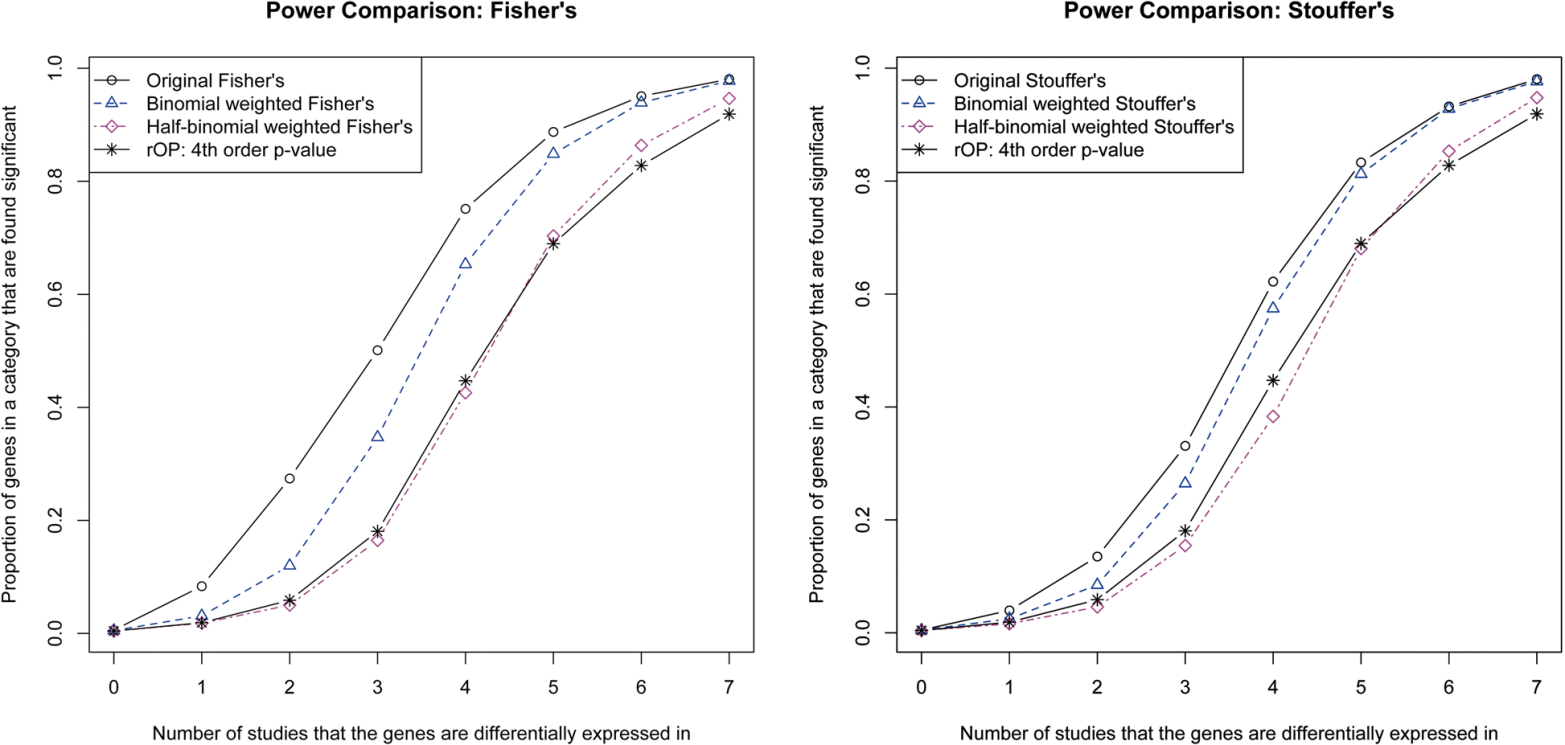
**Comparison of power.** Power comparison for the different methods. The x-axis denotes the 8 categories of genes, categorized by the number of studies that the genes are differentially expressed in. There are 1650 genes in the category 0 (no differential expression in any studies). The rest of the categories contain 50 genes each. For each category, the proportion of genes found significant within that category are plotted for each method.

Figure 2 compares the power of the methods for different categories of genes. Genes are categorized based on the number of studies that they are differentially expressed in (0 to 7). The proportion of genes rejected within each category are plotted for the different methods. Before we compare the methods, it is important to note that the reading of these plots may be different from traditional power plots. Normally when looking at a power plot, the higher the power the better. In our case, however, this is not true for all categories. Remember that our goal is to focus on a particular subset of the non-null space - genes that are differentially expressed in at least 4 studies. Therefore, all non-null genes are not created equal. In particular, rejections of genes in categories 4 through 7 are desirable, whereas rejections of genes in categories 1 through 3 are undesirable. Rejections of genes in category 0 are of course considered false discoveries. As expected, regardless of the method, the proportion of genes rejected within a category increases from 0 to close to 1 as the number of studies that the genes are differentially expressed in increases from 0 to 7 (out of 7). Fisher’s method, being a very powerful method for testing *HS*_1_, has the highest proportion of rejections for every category from 1 to 7. Stouffer’s method also has the highest proportion of rejections when compared to the corresponding weighted versions and the rOP method. However, as mentioned earlier, rejections in categories 0 to 3 are considered undesirable when testing *HS*_4_. Both the WOP methods and the rOP method reject much smaller proportions of genes in categories 1 to 3 compared to the original Fisher’s or Stouffer’s method. Less rejections in categories 1 to 3 come at the expense of less power for categories 4 to 7, which is true for both WOP and rOP methods, but particularly so for the rOP method. For the binomial weighted WOP methods, the rejections for categories 1 to 3 are much lower compared to Fisher’s or Stouffer’s method, but the power for categories 4 to 7 gradually increases to catch up with Fisher’s or Stouffer’s. When it comes to categories 6 and 7, especially category 7, the binomial weighted WOP methods have virtually the same power as Fisher’s and Stouffer’s. On the other hand, the rOP method has the lowest power for categories 6 and 7, which are the genes that are differentially expressed in all or almost all the studies. The half-binomial weighted WOP methods have the lowest rejection rates for categories 1 to 3, even lower than the rOP method, but their power surpasses the power of the rOP method when it comes to categories 6 and 7.

**Figure 3:**
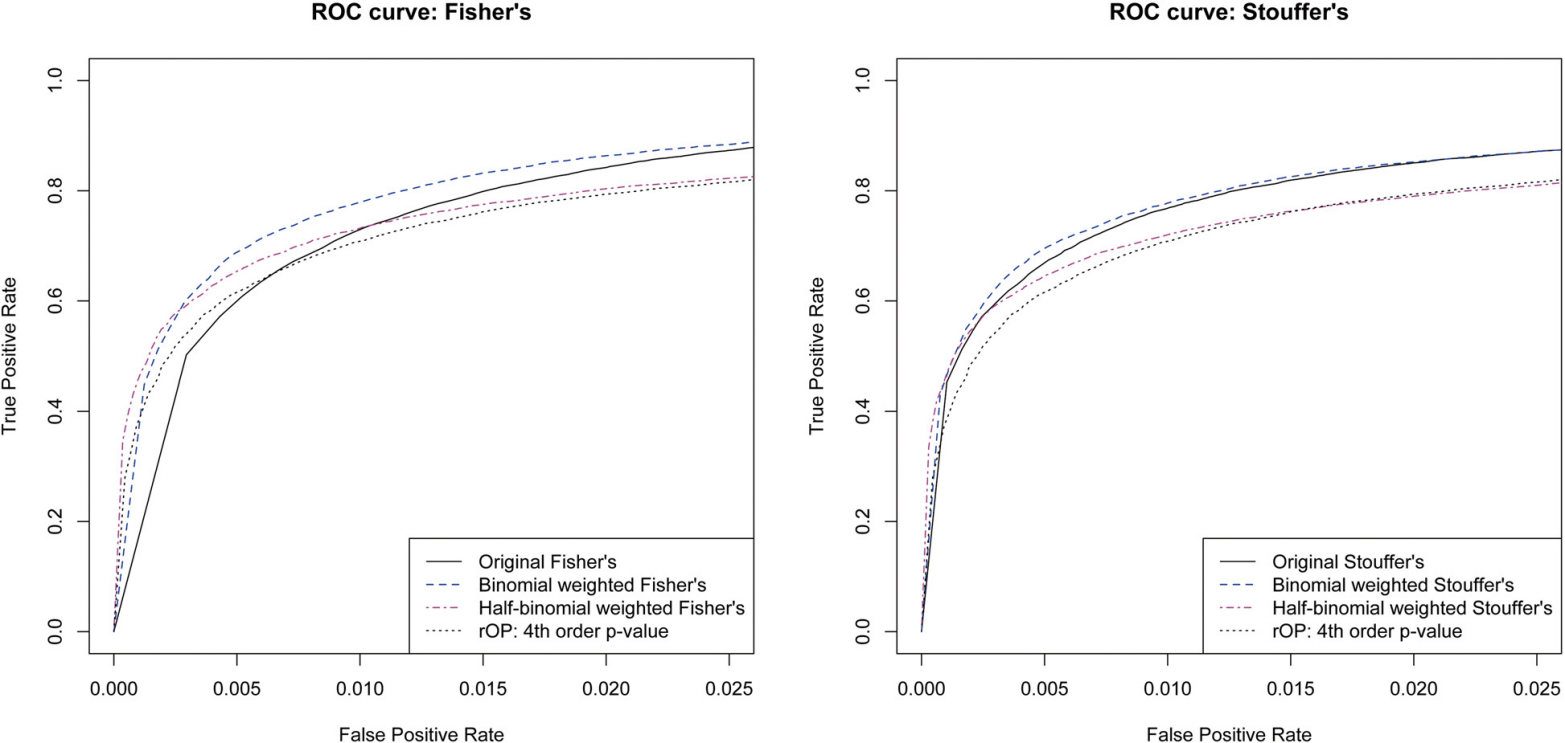
**ROC curves.** ROC curves for the different methods. Rejections of genes differentially expressed in less than 4 studies are considered false positives. Rejections of genes differentially expressed in 4 or more studies are consider true positives.

To better look at the trade off between rejection rates for categories 0 to 3 versus categories 4 to 7, we plotted the ROC curves for the methods, as seen in Figure 3. Since our goal is to test for *HS*_4_, we treat rejections in categories 0 to 3 to be false positives, while rejections in categories 4 to 7 are considered true positives. From the ROC curves we can clearly see that in terms of the trade off between true and false positives the binomial weighted WOP methods beat the original Fisher’s or Stouffer’s method, while the half-binomial weighted WOP methods beat the rOP method. We also gain better insight into the comparison between the binomial and half-binomial weighting schemes. The half-binomial weighting scheme is relatively more conservative, with relatively higher true positive rates at very low false positive rates. On the other hand, the binomial weighting scheme achieves higher power when slightly higher false positive rates are allowed.

In summary, the binomial weighted WOP methods show improvement over the original Fisher’s or Stouffer’s method for testing differential expression in a majority of studies, with lower rejection rates for genes that are differentially expressed in a small number of studies, and just as high power for genes that are differentially expressed in almost all studies. On the other hand, the half-binomial weighted WOP methods are more robust versions of the rOP method, having similar properties to the rOP method, but even lower rejections rates for categories 1 to 3 and higher power for categories 6 and 7. In practice, the binomial weighting scheme is recommended if the user wishes to have a larger pool of significant genes, and when the control of false positives is relatively less important. For better false positive control, the half-binomial weighting scheme is recommended. We note that because of our hypothesis setup, false positives are not the same as type I error. A type I error would be rejecting a gene that is not differentially expressed in any studies. On the other hand, a false positive would be rejecting a gene that is differentially expressed in less than *r* studies.

### An application to meta-analysis of a set of stem cell studies

As an application of the proposed methodology, we conduct meta-analysis on a set of microarray data studies from four stem cell papers: Chin et al. [21], Guenther et al. [22], Newman and Cooper [23] and Chin et al. [24]. We wish to find probesets that are differentially expressed between human induced pluripotent stem (hiPS) cells and human embryonic stem (hES) cells in the majority of studies. Some of the studies contain other samples such as human fibroblasts, but we only used samples from hiPS cells and hES cells. We included studies that had at least two samples for each group (hiPS and hES), giving us a total of 9 studies. All the studies used the Affymetrix Human Genome U133 Plus 2.0 Array platform, which contains 54675 probesets. We directly used the data preprocessed by the original contributors and did not perform any additional normalization, except for taking the log for data that were not already on the log2 scale. We performed a two sample T-test between the hiPS cell samples and the hES cell samples to obtain the original p-values for differential expression for each probeset and each study. The hypothesis setting for the meta-analysis is *HS*_5_, i.e. we aim at testing the alternative that the probeset is differentially expressed in at least 5 out of the 9 studies. We applied the proposed WOP methods, in particular the binomial and half-binomial weighted Fisher’s and Stouffer’s statistic to the p-values. The p-values for the WOP statistics are obtained by comparing the statistics to the corresponding numerical distributions. We also applied the original Fisher’s and Stouffer’s method, and the rOP method (in this case the 5th ordered p-value). The p-values for the rOP method are obtained via its theoretical distribution. To adjust for multiple testing, we applied the Benjamini-Hochberg (1995) procedure afterwards, controlling the false discovery rate at the 0.05 level.

**Figure 4:**
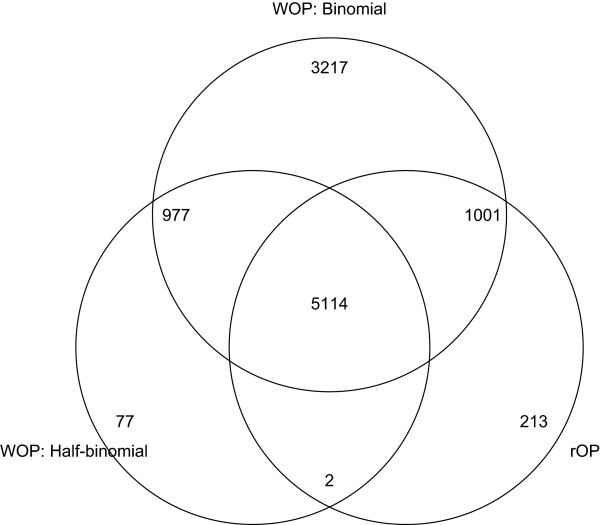
**Venn diagram for stem cell studies.** Venn diagram for the probesets found significant by the binomial weighted Fisher’s method, the half-binomial weighted Fisher’s method and the rOP method.

**Figure 5:**
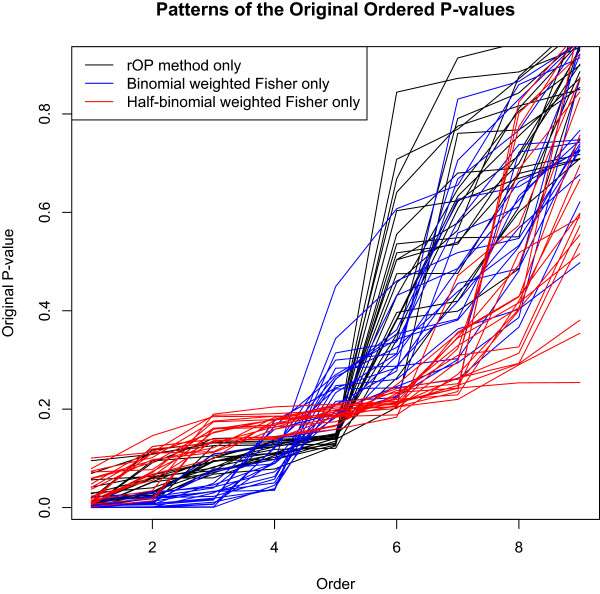
**P-value patterns for different methods.** Pattern of the original ordered p-values from the 9 studies for probesets detected by one of the three methods only. The x-axis is the order of the p-values from the 9 studies. The y-axis is the p-values. The plot includes a random subset of 20 probesets that are detected exclusively by each of the three methods.

The number of probesets significant for the meta-analysis using the different methods are summarized in Table 1. The original Fisher’s and Stouffer’s methods found the most number of significant probesets. It is likely that they picked out many probesets that are differentially expressed in a few, but less than half the studies. The half-binomial weighted WOP methods and the rOP method are relatively more conservative and focused. Figure 4 shows a Venn diagram of the probesets found significant by the binomial and half-binomial weighted Fisher’s method and the rOP method. We can see that the probesets detected by both the half-binomial weighted Fisher’s method and the rOP method are mostly detected by the binomial weighted Fisher’s method as well. However, there are still a number of unique probesets that are only detected by either the half-binomial weighted Fisher’s method or the rOP method. To get an idea of the types of probesets detected by only one of the three aforementioned methods, we randomly selected some of these probesets and plotted the ordered original p-values from the 9 studies for these probesets. As seen in Figure 5, probesets exclusively detected by the binomial weighted Fisher’s method tend to have very small values for the two or three smallest p-values, but relatively larger values starting the 5th ordered p-value. This shows that the binomial weighted Fisher’s method is more prone to influences by the smallest p-values, since it takes into account all the p-values in the statistics. The rOP method, which uses the 5th ordered p-value as the statistic, exclusively identifies probesets that are guaranteed to have relatively small p-values up to the 5th ordered p-value, but tend to have very large values starting the 6th ordered p-value. This shows the sensitivity of the rOP method to the particular value of *r* chosen. On the other hand, the half-binomial weighted Fisher’s method weights in the 5th through the 9th ordered p-values, and thus is able to identify probesets that have relatively small values through larger ordered p-values. In other words, probesets that have relatively small p-values for most of the studies can be exclusively identified by the half-binomial weighted method, even if the smallest p-values are not very small.

**Table 1:** Number of significant probesets for the meta-analysis of stem cell studies by different methods

**Method**	**Fisher’s**	**Stouffer’s**	**rOP**
Original (unweighted)	16508	11969	6330
WOP: Binomial weighted	10309	8927	N/A
WOP: Half-binomial weighted	6170	5805	N/A

To look at the pathways associated with the significant lists of probesets, we performed functional annotation clustering analysis using DAVID (Huang et al, 2009), which is available at http://david.abcc.ncifcrf.gov/home.jsp. Some of the top functions that show up include metal-binding, nucleoplasm, ubl conjugation, vasculature development and head/face development. We also looked at the pathways for the probesets that were exclusively detected by one of the methods. Functions such as neuron projection, neuron differentiation and development, which are meaningful in the stem cell study setting, were found to be associated with the probesets exclusively identified by the half-binomial weighted Fisher’s method. These functions did not show up in pathway analyses of the other lists.

### Further applications and comparisons with the rOP method

To compare the performances of the WOP methods and the rOP method Song and Tseng [1] in real data application, we applied our WOP methods to the three microarray meta-analysis applications in Song and Tseng [1]. The first application consists of comparisons of two subtypes of brain tumors - anaplastic astrocytoma (AA) and glioblastoma multiforme (GBM), from 7 studies. The second application combines 9 studies comparing post-mortem brain tissues between MDD patients and control samples. In the third application, 16 diabetes microarray studies consisting of different organisms and tissues were combined. See Song and Tseng [1] for more details on the contexts of these three meta-analysis applications.

To ensure that the results are directly comparable, for each meta-analysis we directly used the two-sided p-values for each gene and each individual study calculated in Song and Tseng [1]. In Song and Tseng [1], permutation analysis is used for the brain cancer studies and the MDD studies, while theoretical distributions are used to obtain results for the diabetes studies. We follow Song and Tseng [1] and also directly use the two-sided p-values for the permuted datasets provided by Song and Tseng [1] for the brain cancer studies and the MDD studies. See Song and Tseng [1] for more details on the preprocessing of the data and the calculation of the original p-values.

We applied our WOP methods, namely the binomial and half-binomial weighted Fisher’s and Stouffer’s statistic, to the three sets of studies. For the brain cancer studies, we test for *HS*_4_ out of 7 studies. For the MDD studies we test for *HS*_5_ out of 9 studies. For the diabetes studies, we test for *HS*_9_ out of 16 studies. We also applied the corresponding rOP methods for testing the same hypotheses, using *r*=*m*=4, 5 and 9 respectively for the three meta-analyses. In addition, we applied the rOP methods using the selected *r* values in Song and Tseng [1] for comparison. To be specific, Song and Tseng [1] used *r*=5 for the brain cancer studies, *r*=7 for the MDD studies, and *r*=12 for the diabetes studies. Permutation analysis is used for the brain cancer studies and the MDD studies for all methods. For the diabetes studies, we used our numerically simulated distribution to obtain the p-values for the WOP statistics. Table 2 shows the numbers of significant genes found using the different methods for the three meta-analyses with the FDR controlled at the 0.05 level. We can observe that the WOP methods using the binomial weighting scheme generally detects more significant genes than the half-binomial weighting scheme. In most cases, the rOP methods (using either *r*=*m* or selected *r*) detect less genes than the WOP methods, although in general closer in number to the WOP methods using the half-binomial weighting scheme.

**Table 2:** Number of significant genes for the three meta-analyses by different methods

	**WOP Fisher**	**WOP Fisher**	**WOP Stouffer**	**WOP Stouffer**	**rOP**	**rOP**
	**binomial**	**half-binomial**	**binomial**	**half-binomial**	***r*** **=** ***m***	selected r*
Brain Cancer	2477	1887	2261	1805	1921	1469
MDD	1070	930	1152	969	565	617
Diabetes	1333	1016	1277	1004	912	636

^*^For the rOP method, “selected r” refers to the choice of r in Song and Tseng [1] for a particular meta-analysis.

**Figure 6:**
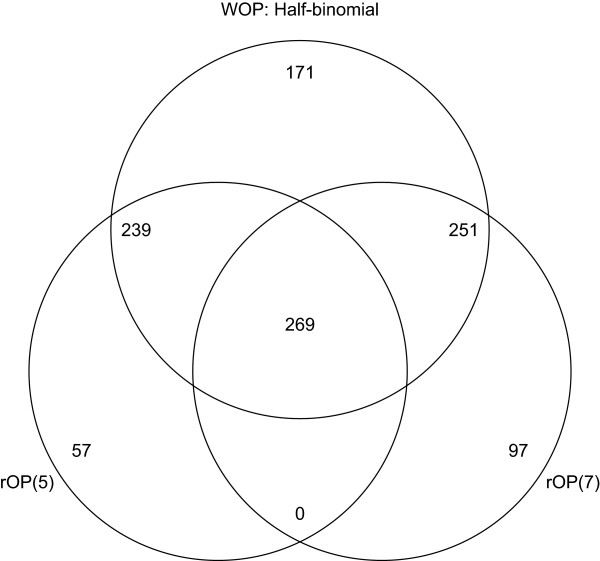
**Venn diagram for MDD studies.** Venn diagram for the genes found significant in the meta-analysis of the MDD studies by the rOP method based on *r*=*m*, the rOP method based on selected *r*, and the half binomial weighted Fisher’s method. In this case, *m*=5 and the selected *r*=7.

One interesting observation is that for the MDD studies, the rOP method based on *r*=*m*=5 detects less genes than based on the selected *r*=7. This result is counterintuitive, since one would expect that genes that are differentially expressed in at least 7 studies would be a subset of the genes that are differentially expressed in at least 5 studies. The result could be due to the fact that r is selected in Song and Tseng [1] to optimize the number of significant genes and therefore outperforms a general choice of *r*=*m*. Nonetheless, this reflects the fact that the rOP method is sensitive to the choice of *r*. To further investigate this problem, we looked at the overlap of the detected genes by the rOP methods using either *r*=*m* or selected *r*, as well as with the detected genes by the half-binomial weighted Fisher’s method. See Figure 6 for a Venn diagram of the genes detected by the aforementioned three methods for the MDD studies. As shown in Figure 6, only 269 genes overlap between the rOP methods with two different choices of *r*, which is less than half of the genes detected by either method. However, all 269 genes are detected by the half-binomial weighted Fisher’s method. In addition, the WOP method also picked up 251 of the genes only detected by rOP based on selected *r* and 239 of the genes only detected by rOP based on *r*=*m*, which accounts for most of the genes detected by either method. Further, we noticed that even for the brain cancer studies and the diabetes studies, where rOP based on *r*=*m* did detect more genes than rOP based on selected *r*, there are still a large number of genes detected by rOP based on selected *r* that are not detected by rOP based on *r*=*m*. On the other hand, most of these genes that are detected by only one of the rOP methods are detected by the half-binomial weighted Fisher’s method.

For comparison, to see how the results would differ for different choices of *r* for the WOP methods, we applied the WOP method to the MDD studies again - this time testing *HS*_7_. We used the half-binomial versions of the three weighting schemes for *m*<*r*≤*K* that were discussed earlier: *w*^*h* *b*1^, *w*^*h* *b*2^ and *w*^*h* *b*3^. We used Fisher’s summary statistic in the WOP method. The numbers of genes found by using the three weighting schemes are 760, 770 and 774 respectively. The three weighting schemes are fairly consistent with each other, since for each weighting scheme more than 90*%* of the genes found were also found by the other two weighting schemes. Comparing to the number of genes found by the half-binomial weighted Fisher’s method for testing *HS*_5_, which is 930, notice that the corresponding WOP methods for testing *HS*_7_ yield smaller numbers, conforming to our expectations. As discussed earlier, the ideal result would be that the genes detected for *HS*_7_ be a subset of the genes detected for *HS*_5_. In reality, over 70*%* of the genes detected by WOP methods for *HS*_7_ were also detected for *HS*_5_ (the percentage being 72.6*%*, 71.4*%* and 74.2*%* for the three weighting schemes respectively). Recall that for the rOP method, only 43.6*%* of genes detected for *r*=7 were also detected for *r*=5. Even though the WOP methods are not perfect, we can still see the great improvement in robustness compared to the rOP method.

In summary, our observations confirm that the results of the rOP method are indeed heavily dependent on the choice of *r*. Whereas our WOP methods show much higher robustness. In particular, the WOP methods for testing *HS*_*m*_ has shown superior robustness by being able to cover most of the genes detected by the rOP methods using different *r*. Since in practice it is not often clear which particular *HS*_*r*_ should be tested, we believe the WOP methods for testing *HS*_*m*_ is a better choice when the goal is to detect signal in the majority of studies.

## Conclusions

Meta-analysis is a useful tool in integrating data from different sources to test a particular hypothesis. While this paper mainly discussed the application of meta-analysis on microarray differential expression studies, other areas of genomic studies have increasingly relied on the use of meta-analysis, such as genome-wide association studies (GWAS). Some seminal studies in this area include Scott et al. [25] and Willer et al. [26]. Meta-analysis is also frequently used in clinical studies, psychological studies and statistical applications in other social sciences. More and more meta-analyses nowadays aim at detecting consistent findings across a number of studies. While most of the traditional meta-analysis methods test for significance in at least one of the studies, it is important to develop new meta-analysis methods that focus on testing for significance in the majority of studies.

The weighted ordered p-value (WOP) method provides such a framework. It is unique in its use of weights that are based on the order of the p-values. The rOP method Song and Tseng [1], which is also based on ordered p-values, can be considered a very special case under the WOP framework, where all the weight is placed on one single ordered p-value. The WOP methods do not require pre-specification of *r* and is less sensitive to the choice of its value. The half-binomial weighted WOP methods have been shown to be more robust and have better receiver operating characteristics compared to the corresponding rOP method.

As pointed out by a reviewer, one of our previously published meta-analysis methods also considers the issue of heterogeneity and utilizes weights. However, these two methods are quite different in concept. The method in [9] applies weights to the genes, essentially to re-rank the genes by adding in information about heterogeneity. On the other hand, the WOP method applies weights to the multiple p-values for each gene. Although, conceptually, we can first apply the WOP method to each gene and then use the method in [9] on top of that to re-rank the genes.

One advantage of the WOP framework is its flexibility. The framework allows for different weighting schemes and summary statistics to be used. Even though this paper mainly focused on two particular weighting schemes based on the binomial distribution and two summary statistics (Fisher’s and Stouffer’s statistics), in general, other summary statistics and weighting schemes can be used. Future research can be done to try to optimize the weighting scheme to suit specific meta-analysis purposes.

## Competing interests

The authors declare that they have no competing interests.

## Authors’ contributions

YL developed the method’s framework, carried out the simulation studies and data analysis, and drafted the manuscript. DG conceived of the weighting schemes, supervised the research process and revised the manuscript. All authors read and approved the final manuscript.

## References

[B1] Song C Tseng GC **Hypothesis setting and order statistic for robust genomic meta-analysis** Ann Appl Stat 2014 8 777 800 10.1214/13-aoas683 PMC4222050 25383132

[B2] Fisher RA Statistical methods for research workers 1925 Oliver and Boyd: Edinburgh

[B3] Stouffer SA Suchman EA Devinney LC Star SA Williams RM The American soldier: adjustment during army life 1949 Princeton, NJ: Princeton University Press

[B4] Choi JK Yu U Kim S Yoo OJ **Combining multiple microarray studies and modeling interstudy variation** Bioinformatics 2003 19 84 90 10.1093/bioinformatics/btg1010 12855442

[B5] Wilkinson B **A statistical consideration in psychological research** Psychol Bull 1951 48 156 158 14834286 10.1037/h0059111

[B6] Phillips D Ghosh D **Testing the disjunction hypothesis using Voronoi diagrams with applications to genetics** Ann Appl Stat 2014 8 801 823

[B7] Benjamini Y Heller R **Screening for partial conjunction hypotheses** Biometrics 2008 64 1215 1222 18261164 10.1111/j.1541-0420.2007.00984.x

[B8] Hong F Breitling R McEntee CW Wittner BS Nemhauser JL Chory J **RankProd: a bioconductor package for detecting differentially expressed genes in meta-analysis** Bioinformatics 2006 22 2825 2827 16982708 10.1093/bioinformatics/btl476

[B9] Li Y Ghosh G **Assumption weighting for incorporating heterogeneity into meta-analysis of genomic data** Bioinformatics 2012 28 807 814 22285559 10.1093/bioinformatics/bts037 PMC3307113

[B10] Olkin I Saner H **Approximations for trimmed Fisher procedures in research synthesis** Stat Methods Med Res 2001 10 267 276 11491413 10.1177/096228020101000403

[B11] Lancaster H **The combination of probabilities: an application of orthonormal functions** Aus J Stat 1961 3 20 33

[B12] Mosteller F Bush RR **Selected quantitative techniques** Handbook of Social Psychology 1954 Cambridge, MA: Addison-Wesley

[B13] Won S Morris N Lu Q Elston R **Choosing an optimal method to combine P-values** Stat Med 2009 28 1537 1553 19266501 10.1002/sim.3569 PMC2771157

[B14] Makambi KH **Weighted inverse chi-square method for correlated significance tests** J Appl Stat 2003 30 225 234

[B15] Li J Tseng GC **An adaptively weighted statistic for detecting differential gene expression when combining multiple transcriptomic studies** Ann Appl Stat 2011 5 994 1019

[B16] Xie M Singh K Strawderman WE **Confidence distributions and a unifying framework for meta-analysis** J Am Stat Assoc 2011 106 320 333

[B17] Zaykin DV **Optimally weighted Z-test is a powerful method for combining probabilities in meta-analysis** J Evol Biol 2011 8 1836 1841 21605215 10.1111/j.1420-9101.2011.02297.x PMC3135688

[B18] Benjamini Y Hochberg Y **Controlling the false discovery rate: a practical and powerful approach to multiple testing** J R Stat Soc Series B 1995 57 289 300

[B19] Pearson K **On a new method of determining “goodness of fit”** Biometrika 1934 26 425 442

[B20] Owen AB **Karl Pearson’s meta-analysis revisited** Ann Stat 2009 37 3867 3892

[B21] Chin MH Mason MJ Xie W Volinia S Singer M Peterson C Ambartsumyan G **Induced pluripotent stem cells and embryonic stem cells are distinguished by gene expression signatures** Cell Stem Cell 2009 5 111 123 19570518 10.1016/j.stem.2009.06.008 PMC3448781

[B22] Guenther MG Frampton GM Soldner F Hockemeyer D Mitalipova M Jaenisch R Young RA **Chromatin structure and gene expression programs of human embryonic and induced pluripotent stem cells** Cell Stem Cell 2010 7 249 257 20682450 10.1016/j.stem.2010.06.015 PMC3010384

[B23] Newman AM Cooper JB **Lab-specific gene expression signatures in pluripotent stem cells** Cell Stem Cell 2010 7 258 262 20682451 10.1016/j.stem.2010.06.016

[B24] Chin MH Pellegrini M Plath K Lowry WE **Molecular analyses of human induced pluripotent stem cells and embryonic stem cells** Cell Stem Cell 2010 7 263 269 20682452 10.1016/j.stem.2010.06.019 PMC3276111

[B25] Scott LJ Mohlke KL Bonnycastle LL Willer CJ Li Y Duren WL Erdos MR Stringham HM Chines PS Jackson AU Prokunina-Olsson L Ding CJ Swift AJ Narisu N Hu T Pruim R Xiao R Li XY Conneely KN Riebow NL Sprau AG Tong M White PP Hetrick KN Barnhart MW Bark CW Goldstein JL Watkins L Xiang F Saramies J **A genome-wide association study of type 2 diabetes in Finns detects multiple susceptibility variants** Science 2007 316 1341 1345 17463248 10.1126/science.1142382 PMC3214617

[B26] Willer CJ Sanna S Jackson AU Scuteri A Bonnycastle LL Clarke R Heath SC Timpson NJ Najjar SS Stringham HM Strait J Duren WL Maschio A Busonero F Mulas A Albai G Swift AJ Morken MA Narisu N Bennett D Parish S Shen H Galan P Meneton P Hercberg S Zelenika D Chen WM Li Y Scott LJ Scheet PA **Newly identified loci that influence lipid concentrations and risk of coronary artery disease** Nat Genet 2008 40 161 169 18193043 10.1038/ng.76 PMC5206900

